# Significance of Serum Alpha-Glutathione S-Transferase Assessment in Hepatitis C Patients with Different Alanine Aminotransferase Patterns

**DOI:** 10.4021/gr269w

**Published:** 2011-01-20

**Authors:** Samir M Abdel-Moneim, Hamdy Sliem

**Affiliations:** a Departments of Biochemistry, Faculty of Medicine, Suez Canal University, Ismailia, Egypt; b Internal Medicine, Faculty of Medicine, Suez Canal University, Ismailia, Egypt

**Keywords:** Hepatitis C, Glutathione transferase

## Abstract

**Background:**

Alpha-Glutathione S-transferase (α-GST) is a liver enzyme which showed properties making it useful in assessment of liver cell damage. A number of studies demonstrated its early elevation in different hepatic insults, but its pattern in HCV was controversial. Consequently, we planned this work to study the significance of Serum Alpha-Glutathione S-Transferase (α-GST) assessment in hepatitis C patients with different alanine aminotransferase (ALT) patterns.

**Methods:**

Sixty-five untreated male patients with history of hepatitis C virus (HCV) positive antibodies and 21 healthy age- and sex-matched control subjects were enrolled in this study. Sera were collected for confirmation of the presence of HCV antibodies (by ELISA) as well as for assessment of the levels of α-GST, ALT, aspartate aminotransferase, alkaline phosphatase, gamma glutamyl-transferase, total proteins, albumin and HCV RNA. HCV RNA was detected by the reverse transcription polymerase chain reaction (RT-PCR). Based on ALT level, patients were divided into three groups. Twelve patients with normal ALT levels (NALT), 29 with near normal ALT levels (NNALT), and 24 with high ALT levels (HALT). All data were statistically analyzed for significance and correlation as well as sensitivity, specificity, positive and negative predictive values.

**Results:**

The mean value of α-GST in HCV patients was significantly higher compared to the control with 82% sensitivity, 85% specificity, 98% positive predictive value and 63% negative predictive value. These results were more or less similar to the results of ALT and higher than the results of all the other assayed liver function tests. The sensitivity, and positive and negative predictive values of α-GST were lower than aminotransferases, but higher than the other assayed liver function tests in NNALT and HALT groups. Nevertheless, in NALT, these parameters were higher for α-GST than all the other assayed liver function tests including aminotransferases.

**Conclusions:**

Assay of α-GST has an adjuvant in evaluation of liver cell damage in HCV patients. However, its role is much more valuable in patients with normal aminotransferases for early detection of liver cell damage.

## Introduction

Hepatitis C virus (HCV) is a critical factor of liver disease and one of the most important health issues worldwide [1]. HCV infection showed serious complications such as liver cirrhosis and liver cell failure as well as hepatocellular carcinoma. These complications usually occur after about 20 years of infection [2], which may give a room for detection and proper management and follow up of hepatocellular damage. Nevertheless, this may need a sensitive and accurate parameter for early detection of liver damage. Although assessment of plasma level of alanine aminotransferase (ALT) is known to be the most specific parameter for hepatocellular damage, it is found to be elevated only in 40.2% of HCV seropositive patients [3] and 54.4% of HCV RNA positive patients [4]. Moreover, aminotransferase levels in those patients do not reflect HCV-RNA levels [5, 6].

Alpha-Glutathione S-Transferase (α-GST) is an enzyme responsible for cellular detoxifying processes. Its wide hepatic distribution, high cytosolic concentration, and short plasma half-life make its monitoring more useful than conventional biochemical liver function tests as a marker of hepatocellular damage [7]. Immunohistochemical studies have demonstrated that α-GST present in the liver is localized exclusively in hepatocytes and its plasma activity is reported to reflect liver damage better than that of aminotransferases [8].

Regarding the HCV infection, controversial data were demonstrated. Namour et al showed low sensitivity of α-GST for cytolysis of hepatocytes [4], while Federico et al and Helaly and Mahmoud described α-GST as a more useful marker than other biochemical parameters in assessment of hepatocellular damage in this condition [7, 9]. Consequently, our study was planned to investigate the significance of assessment of α-GST plasma levels in HCV infection with different ALT patterns and its relation to viremia and other biochemical parameters of hepatocellular damage.

## Patients and Methods

Sixty-nine untreated male patients (aged 27 - 56 years, mean 42.3 ± 9.2) with a history of seropositive HCV antibodies from the attendants of the outpatient clinic of the Suez Canal University Hospital were enrolled in this study. Twenty-one healthy male adults matched for age were considered as a control group. All had negative history of Hepatitis B and Bilharziasis. After obtaining written consents from all patients and controls, 5 ml of morning blood sample were collected in plain Vacutainer tubes to separate sera for the following: (1) Confirmation of the presence of HCV antibodies by the third generation enzyme-linked immunosorbent assay (ELISA) using the Abbott Murex anti-HCV (version 4.0) kit provided by Murex Biotec S.A. Ltd, Kyalami, Republic of South Africa. (2) Estimation of the serum level of alanine aminotransferase (ALT), aspartate aminotransferase (AST), alkaline phosphatase (ALP), gamma glutamyl-transferase (GGT), total proteins (TP), and albumin (Alb) by the automated Hitachi 912 using adaptations of the methods recommended by the International Federation of Clinical Chemistry. (3) Detection of HCV-RNA by the reverse transcription polymerase chain reaction where RT-PCR was done using TaqMan® One-Step RT-PCR Master Mix Reagents Kit (Cat No. 4309169, Applied Biosystems, Foster City, USA), primers and a FAM labeled HCV probe for the 5′ non coding region of the HCV genome (Applied Biosystems, Foster City, USA). An exogenous internal control (internal quality marker) was included with each sample to assess the efficiency of the procedure. The lower limit of detection is 100 IU/ml. (4) Determination of α-GST by the ELISA HEPKIT provided by Biotrin International, Dublin, Ireland, according to the manufacturer’s instructions and as suggested by the manufacturer and the literature where α-GST levels ≤ 8 ng/ml were considered normal [10, 11].

### Statistical Analysis

The data were coded and organized. The final study results were stated using the SPSS program version 14. Results were presented through Tables. Student’s *t-test*, correlation coefficient, and Chi-square test were used to evaluate the results. Chi-square test was used for qualitative variables, while independent *t-*test was used for quantitative variables. Correlation analysis was performed using Pearson's test. The sensitivity, specificity, positive and negative predictive values of each parameter were calculated according to the formulae described by Zhou et al [12]. Statistical significance was considered at *P*-value < 0.05 and highly significance at *P*-value < 0.001.

## Results

ELISA technique for assessment of HCV antibody levels showed results above the cut off value recommended by the manufacturer in sixty-five patient samples and below the cut off value in all control samples. The four patients with seronegative HCV antibodies were excluded from the study. Seropositive HCV patients (All HCV) were subdivided according to the suggestion of Giannini et al into three groups depending on the serum level of ALT [13]. The first group contained twelve patients with ALT below the high limit of the reference value, < 40 IU/L, (normal ALT group; NALT); the second contained twenty nine patients with ALT level above the high limit of the reference value and below double the high limit of the reference value, 40 - 80 IU/L, (near normal ALT group; NNALT); and the third contained twenty four patients with ALT level more than double the high limit of the reference value, > 80 IU/L, (high ALT group; HALT). Calculation of the positive predictive value of HCV RNA, ALT, AST, ALP, GGT, TP, Alb, and α-GST showed 100% specificity for all these parameters except α-GST which was 98.15%. The results are shown in Table 1-3.

**Table 1:** Comparison Between the Mean ± SD of HCV RNA, ALT, AST, ALP, GGT, TP, Alb, and α-GST Levels in the Study Groups.

	Control (*n* = 21)	NALT (*n* = 12)	NNALT (*n* = 29)	HALT (*n* = 24)	All HCV (*n* = 65)
HCV RNA (IU/ml (x10^4^)) P	0.00	1.56 ± 2.8 0.000	16.71 ± 23.3 0.000	32.38 ± 40.5 0.000	19.70 ± 30.9 0.000
ALT (IU/L) *P*	25.29 ± 7.1	30.08 ± 5.8 0.514	61.45 ± 11.5 0.028	132.42 ± 53.3 < 0.001	81.86 ± 52.3 < 0.001
AST (IU/L) *P*	24.26 ± 6.6	27.75 ± 5.1 0.384	45.45 ± 9.5 0.035	97.92 ± 46.7 < 0.001	61.55 ± 40.7 < 0.001
ALP (IU/L) *P*	164.52 ± 40.9	171.25 ± 40.3 1.000	191.62 ± 41.2 0.991	225.17 ± 65.1 0.040	200.25 ± 54.5 < 0.001
GGT (IU/L) *P*	32.86 ± 8.2	34.42 ± 8.5 0.872	47.69 ± 14.6 0.048	82.21 ± 40.7 < 0.001	57.99 ± 32.8 < 0.001
TP (g/dl) *P*	7.40 ± 0.5	7.10 ± 0.5 0.920	6.69 ± 0.6 0.521	6.52 ± 0.5 0.972	6.71 ± 0.6 0.602
Alb (g/dl) *P*	4.61 ± 0.6	4.23 ± 0.6 0.947	3.86 ± 0.5 0.607	3.45 ± 0.5 0.246	3.78 ± 0.6 1.038
α-GST (ng/ml) *P*	0.27 ± 2.1	10.51 ± 3.4 0.019	16.25 ± 7.7 < 0.001	18.56 ± 9.7 < 0.001	16.04 ± 8.4 < 0.001

P: comparison between various patients groups and control. NALT: normal ALT group (ALT < 40 IU/L); NNALT: near normal ALT group (40 - 80 IU/L); HALT: high ALT group (ALT > 80 IU/L); α-GST: Alpha-Glutathione S-transferase; n: number; ALT: alanine aminotransferase; AST: aspartate aminotransferase; ALP: alkaline phosphatase; GGT: gamma glutamyl-transferase; TP: total proteins; Alb: albumin; HCV: hepatitis C virus.

**Table 2:** Correlation Analysis Between α-GST and HCV RNA, ALT, AST, ALP, GGT, TP, and Alb Levels in the Study Groups.

		HCV RNA	ALT	AST	ALP	GGT	TP	Alb
NALT	r	0.75	0.686	0.269	0.5005	0.562	- 0.322	- 0.265
	*P*	< 0.05	< 0.05	> 0.05	> 0.05	> 0.05	> 0.05	> 0.05
NNALT	r	0.523	0.763	0.737	0.499	0.698	- 0.443	- 0.419
	*P*	< 0.05	< 0.01	< 0.01	> 0.05	< 0.01	> 0.05	> 0.05
HALT	r	0.532	0.909	0.811	0.698	0.773	- 0.495	- 0.502
	*P*	< 0.01	< 0.01	< 0.01	< 0.01	< 0.01	> 0.05	> 0.05
ALL	r	0.645	0.685	0.657	0.549	0.594	- 0.443	- 0.419
HCV	*P*	< 0.01	< 0.01	< 0.01	< 0.01	< 0.01	> 0.05	> 0.05

NALT: normal ALT group (ALT < 40 IU/L); NNALT: near normal ALT group (40 - 80 IU/L); HALT: high ALT group (ALT > 80 IU/L); α-GST: Alpha-Glutathione S-transferase; n: number; ALT: alanine aminotransferase; AST: aspartate aminotransferase; ALP: alkaline phosphatase; GGT: gamma glutamyl-transferase; TP: total proteins; Alb: albumin; HCV: hepatitis C virus.

**Table 3:** Sensitivity, Specificity, Positive Predictive Value and Negative Predictive Value of HCV RNA, ALT, AST, ALP, GGT, TP, Alb, and α-GST in all the HCV Patients Groups and Control.

	Sensitivity %	Specificity %	+ ve predictive	- ve predictive
			Value%	Value%
**HCV All Patients (*n* = 65)**				
HCV RNA	70.77	77.91	100.00	52.50
ALT	81.54	86.05	100.00	63.64
AST	64.62	73.26	100.00	47.73
ALP	9.23	31.40	100.00	26.25
GGT	46.15	59.30	100.00	37.50
TP	12.31	33.72	100.00	26.92
Alb	33.85	50.00	100.00	32.81
α-GST	81.54	84.88	98.15	62.50
**NALT Group (*n* = 12)**				
HCV RNA	41.67	78.79	100.00	75.00
ALT	0.00	63.64	0.00	63.64
AST	0.00	63.64	0.00	63.64
ALP	0.00	63.64	0.00	63.64
GGT	0.00	63.64	0.00	63.64
TP	0.00	63.64	0.00	63.64
Alb	0.00	63.64	0.00	63.64
α-GST	66.67	84.85	88.89	83.33
**NNALT Group (*n* = 29)**				
HCV RNA	75.8	86.00	100.00	75.00
ALT	100.00	100.00	100.00	100.00
AST	62.07	78.00	100.00	65.63
ALP	0.00	42.00	0.00	42.00
GGT	41.38	66.00	100.00	55.26
TP	13.79	50.00	100.00	45.65
Alb	27.59	58.00	100.00	50.00
α-GST	79.31	86.00	95.83	76.92
**HALT Group (*n* = 24)**				
HCV RNA	79.17	88.89	100.00	80.77
ALT	100.00	100.00	100.00	100.00
AST	100.00	100.00	100.00	100.00
ALP	25.00	60.00	100.00	53.85
GGT	75.00	86.67	100.00	77.78
TP	16.67	55.56	100.00	51.22
Alb	58.33	77.78	100.00	67.74
α-GST	91.67	93.33	95.65	90.91

n = number of cases.

## Discussion

Hepatitis C virus infection is usually monitored by ALT level. However, accumulating data indicate that ALT is not always correlated with liver cell damage, disease progression, or the response of HCV to therapy [14]. Moreover, many HCV RNA positive subjects with normal aminotransferases levels have significant liver damage despite normal liver biochemistry [15]. For accurate evaluation of the liver status in chronic HCV infection, there must be regular liver biopsy that is an aggressive invasive procedure and potentially associated with severe complications [16].

On the other hand, GST system is indirectly involved in hepatocellular damage due to HCV [8]. A significant increase of α-GST level in HCV patients was reported by Helaly and Mahmoud who showed a significant increase of α-GST levels above the cut off value in 91.2% of HCV patients compared to 4.4% for ALT, 17.6% for AST, and 41.2 % for GGT [7]. Vaubourdolle et al reported that the sensitivity was 58%, 68%, and 64% for ALT, AST, and α-GST [17], while Nelson et al reported a sensitivity of 92%, 72%, and 93% for ALT, AST, and α-GST respectively [18].

These data are not far from our results where we found a significant increase in the blood level of α-GST in all HCV group compared to the control group. Moreover, the sensitivity of α-GST was similar to the sensitivity of ALT (81.54%) in all HCV group and higher than the sensitivities of viremia, AST, ALP, GGT, TP, and Alb. In addition, the specifity of α-GST (84.88%) was not far from the specificity of ALT (86.05%) and higher than the specificities of viremia, AST, ALP, GGT, TP, and Alb. Although the positive predictive value of α-GST (98.15%) was lower than all the other assayed parameters, it was not far from the maximum. The negative predictive value of α-GST was more or less similar to ALT and higher than all the other assayed parameters. However, the positive predictive value of α-GST (95.24%) was slightly lower than all the other assayed parameters that were 100%.

Correlation analysis showed significant positive correlation between α-GST and ALT, AST, ALP, and GGT which may indicate a common origin for these enzymes in such cases. A significant positive correlation was detected between α-GST and viral load which may be supported by the study of Giannini et al who showed significantly high HCV RNA load in patients with high α-GST [13].

When we divided the HCV patients into three groups according to the level of ALT, the first group of patients with normal ALT (NALT) showed a significant increase in the α-GST level compared to the control (P < 0.02). Elevation of plasma level of α-GST may be an early indicator of liver damage. Different studies considered the plasma level of α-GST is an actual indicator of the integrity of the hepatocyte [19] or biomarker of liver damage, for example, in hepatic ischemia/reperfusion injury after liver resection [20], cases of rejection of liver transplant [21], liver damage after hepatectomy [22], biliary atresia [23], intrahepatic cholestasis of pregnancy [24], acute drug-induced hepatotoxicity [25], subclinical hepatic injury after anesthesia [26], hepatocellular damage during polymicrobial sepsis [27], or in patients with cystic fibrosis [28] and in chronic alcohol abusers [29].

The sensitivity and specificity of α-GST in NALT group were higher than the sensitivity and specificity of ALT, AST, ALP, GGT, TP, and Alb. Except for viremia, the positive predictive value of α-GST was higher than all the other assayed parameters. The negative predictive value of α-GST was also higher than all the other assayed parameters including viremia. These data may indicate that α-GST may be more valuable than the other assayed parameters in evaluation of liver damage in patients with normal ALT, also, in cases where it is not possible to discriminate between "healthy" carriers and subjects with chronic liver damage, unless liver biopsy is performed. Nevertheless, the indication of liver biopsy in these conditions is not justified where authors considered it unethical to perform liver biopsies in patients with normal liver function tests [13]. Detection and quantitation of the HCV RNA viral load were considered by Puoti et al as an indicator of liver biopsy in these conditions, but they found HCV RNA quantitation is not a useful indicator in clinical practice [15]. Moreover, Engel et al concluded that the intermittent pattern of viremia in HCV patients reduces its value for assessment of HCV infection [30]. On the other hand, we found significant positive correlation between α-GST and viral load within this group. This may support the role of α-GST as an indicator of liver biopsy in HCV patients when the other biochemical parameters are normal.

A significant increase of α-GST serum level was found also in NNALT compared to the control group. A significant positive correlation was found between α-GST and ALT, AST, and GGT within NNALT group that may support the relation of α-GST with the other liver function tests. The sensitivity of α-GST in this group (79.31%) was lower than the sensitivity of ALT which showed 100% sensitivity, but it was higher than the sensitivity of AST, ALP, GGT, TP, and Alb. Specificity of α-GST (86%) was found to be also lower than ALT (100%) but higher than the specificity of AST, ALP, GGT, TP, and Alb. Although α-GST showed lower positive predictive value (95.83%) than most of other investigated parameters, it had higher negative predictive value than all the other parameters except ALT. These data may indicate that assessment of α-GST is not superior to ALT in evaluation of liver damage in NNALT group. However, it may be related to viremia where a significant positive correlation is found between α-GST and viral load. Consequently, assay of α-GST in such patients may be used as an adjuvant with other liver function tests for evaluation of HCV infection.

HALT patients showed also elevation of α-GST serum level which was significant compared to the control (P < 0.001). Comparing the sensitivity and specificity of α-GST with viremia of these patients, we found higher sensitivity and specificity of α-GST (91.67% and 93.33% respectively) than viremia (79.17% and 88.89% respectively). This may be attributed to the intermittent pattern of viremia [30-32]. However, the sensitivity of α-GST in this group (91.67%) was lower than the sensitivity of ALT and AST which showed 100% sensitivity and higher than the sensitivity of ALP, GGT, TP, and Alb. Specificity of α-GST (93.33%) was also lower than ALT (100%) and AST (100%) but higher than the specificity of ALP, GGT, TP, and Alb. The positive predictive value of α-GST (95.65%) was lower than most of all the other investigated parameters. The negative predictive value was also lower than ALT and AST but higher than all the other parameters. These data may reduce the value of assessment of α-GST compared to aminotransferases in patients with high ALT. However, the positive correlation that was found between α-GST and ALT, AST, ALP, GGT, and viral load within this group may give a role for assay of α-GST as a confirmatory test for hepatocellular damage in these patients.

In conclusion, elevations of α-GST serum level in HCV patients with 81.54% sensitivity, and 84.88% specificity may give a role for its assessment in them. It may be assayed for confirmation of liver cell damage. However, its role would be much more valuable in early detection of liver damage in HCV patients with normal ALT where α-GST showed 66.67% sensitivity and 84.85% specificity compared to 0.0% sensitivity and 63.64% specificity for aminotransferases and other investigated liver function tests. It may be an indicator of liver biopsy in such conditions. The main limitation of the study is the relatively small sample size. Further research will be needed to confirm whether these results generalize to general population through a large community based study. In addition, further studies are needed to correlate any liver histopathological changes with serum levels of α-GST in cases of HCV with normal aminotransferases.
